# The witching week of herding on bitcoin exchanges

**DOI:** 10.1186/s40854-021-00323-4

**Published:** 2022-03-07

**Authors:** N. Blasco, P. Corredor, N. Satrústegui

**Affiliations:** 1 Department of Accounting and Finance, Faculty of Economics and Business Administration, University of Zaragoza and Research Institute on Employment, Digital Society and Sustainability (IEDIS), Gran Vía 2, 50005 Zaragoza, Spain; 2 Department of Business Administration, Institute for Advanced Research in Business and Economics (INARBE), Public University of Navarre (UPNA), Campus de Arrosadia s/n, 31006 Pamplona, Spain; 3 Department of Accounting and Finance, Faculty of Economics and Business Administration, University of Zaragoza, Gran Vía 2, 50005 Zaragoza, Spain

**Keywords:** Herding, Expiration effect, Bitcoin, Futures, Exchanges, Intraday data

## Abstract

This paper analyses the herding behaviour among exchanges around the expiration of bitcoin futures traded on the Chicago Mercantile Exchange (CME). The database extends from December 2017 to October 2020, taking as a reference the main exchanges that trade bitcoin (Binance, Bitfinex, Bitstamp, Coinbase, itBit, Kraken, and Gemini) and using hourly closing prices and trading volumes in bitcoin and US dollars. Adapting the proposal of Chang, Cheng and Khorana (2000) (CCK) to test conditional herding, we obtain results that indicate that the herding effect is significant during the week before expiration. After expiration, the herding effect lasts for a few hours and disappears. Information overload originating, among other causes, from sophisticated investors’ strategies may generate this mimetic behaviour. The results show the relevance of intraday data applied to specific events such as expiration since the unconditional analysis shows, in general, anti-herding behaviour throughout the period of study.

## Introduction

Making decisions is like speaking prose - people do it all the time knowingly or unknowingly

Kahneman and Tversky (1984).

Herding is one of the consequences of decision-making. In financial markets, herding occurs when some investors decide to set aside their beliefs and opinions and imitate the decisions of other investors who are thought to be better informed (Scharfstein and Stein 1990). In a context of bounded rationality, when individuals’ private information is overwhelmed by the influence of public information, many investors may tend to follow the market consensus (Devenow and Welch 1996; Bikhchandani and Sharma 2001; Hirshleifer and Teoh 2003, among others, argue along these lines).

The herding effect has been studied from several perspectives (activity sectors, types of investors, analysts, asset characteristics, etc.) and in different markets. Spyrou (2013) offers an interesting survey of papers on herding. In recent years, however, the boom in cryptocurrencies has opened a new field of study regarding this behavioural phenomenon since cryptocurrencies offer a new informational framework that presumably differs from that of more traditional financial markets.

The underlying technology, i.e., blockchain, the information provided by the social networks that are attracting growing interest in these new products, and the information generated within crypto-exchanges themselves allow investors to have an apparently more complete set of information that is not as easily available for other financial products. Corbet et al. (2019) provide a systematic review of the characteristics of cryptocurrencies. Kou et al. (2021a) gather some studies focused on the importance of fintech investment in blockchain systems for sustainable economic development and the simplification and recording of financial operations, among other advantages. Although all these features should contribute to greater informational transparency, both the lack of clear international regulation and the emergence of information overload may undermine such transparency.

The purpose of this paper is to analyse the herding intensity among spot bitcoin exchanges at a very specific moment: around the expiration time of bitcoin futures, when the information flow can noticeably change. The motivation for this study derives from the fact that herding, particularly in the cryptocurrency market, has become a key topic in behavioural finance, as well as the expectations raised by the creation of regulated bitcoin futures in December 2017.

Following the frequently used proposal of Chang et al. (2000) (CCK), we expand their model to analyse the herding effect around the expiration date of the bitcoin futures contracts traded in the Chicago Mercantile Exchange (CME). That is, we aim to determine whether there is herding behaviour conditioned by the event of expiration. For the analysis, we consider the main bitcoin exchanges worldwide during the period 2018–2020. This paper contributes to the existing literature from both the herding perspective and the cryptocurrency perspective. First, to the best of our knowledge, this study represents the first time that the herding effect has been analysed among exchanges around the futures expiration time. We think that this novelty is valuable since herding among bitcoin exchanges has been studied recently only by Blasco and Corredor (2021). Their analysis was carried out by distinguishing between large and small exchanges and using daily data. In this paper, we use intraday data from the most significant international exchanges. The previous literature highlights the importance of using this data frequency to detect behavioural biases more accurately. Intraday data enable us to find behavioural patterns that can be masked in daily data. The paper analyses imitative behaviour from both the unconditional perspective and the perspective of being conditioned by the specific expiration time, given that there may be differential behaviour by investors before and after the precise expiration time.

Second, this study provides added value to the literature on cryptocurrencies. There have been some studies on herding behaviour among different cryptocurrencies (Bouri et al. 2019; da Gamma Silva et al. 2019; Kallinterakis and Wang 2019; Stavroyiannis and Babalos 2019; Vidal-Tomás et al. 2019; Ballis and Drakos 2020; Kaiser and Stöckl 2020; Kyriazis 2020 or Raimundo Júnior et al. 2020) and its relationship with some special events, such as the COVID-19 pandemic (Yarovaya et al. 2021) and informative signals (Philippas et al. 2020). However, none of these studies has focused on herding among exchanges around futures expiration.

Third, we think that our paper contributes to the financial literature on herding in more traditional markets. Although herding behaviour has been detected previously in a number of markets and assets (Chen 2013, or Chiang and Zheng 2010, among others), herding on the expiration date has been studied only in some assets traded in the Spanish market (Blasco et al. 2010). Our current analysis can offer additional knowledge, as it considers different international exchanges trading the same asset, allowing arbitrage strategies in a global market that trades 24/7. These strategies are particularly sought after around the futures expiration times. Under this framework, the herding effect may appear differently from that in more traditional markets and assets. The information flow in the cryptocurrency market may have distinct characteristics that cause peculiar patterns in investors’ imitative behaviour.

The rest of the paper is structured as follows: the next section describes the theoretical framework and the working hypotheses; section three describes the database; the fourth section presents the methodology; the fifth and sixth sections contain the results and the robustness analysis; and, finally, the last section summarizes the main conclusions obtained.

## Theoretical framework and hypotheses

Behavioural finance tries to explain how investors make decisions in a context of bounded rationality, making the efficient market hypothesis compatible with some empirical regularities found in financial markets. Within this framework, herding behaviour has aroused special interest over the last three decades. Among the variety of reasons explaining herding behaviour in financial markets, we note reputation costs (Scharfstein and Stein 1990, or Trueman 1994), the activity sector to which a company belongs (Demirer and Zhang 2018), and even some variables associated with the quality of a specific informational environment (Chang and Lin 2015 or Blasco et al. 2017). Nevertheless, investors’ reaction to the arrival of information is a common key aspect in all of them. Since the seminal papers by Sheleifer and Summers (1990), Hirshleifer et al. (1994) and Devenow and Welch (1996), mimetic behaviour has been related to either similar reactions of investors to an information set or the lack of quality information. The latter induces contagion in decisions when investors acquire (noisy) information by observing the actions of other agents (Bikhchandani et al. 1992; Welch 1992). More recently, Demirer et al. (2019) suggest a connection between herding and flash events, observing that information in particularly extreme moments with sudden price fluctuations can cause mimetic behaviour. However, as Li et al. (2021) indicate, financial data are social data dominated by multiple complicated latent factors, and they can be affected by changing social environments and time. Therefore, it is difficult to find a catalogue of the circumstances under which herding may appear, which means that herding can be analysed from different perspectives. For example, Zha et al. (2020) review the application of opinion dynamics in finance and business. From the perspective of opinion dynamics, herding can arise if the final evolution of opinion tends towards consensus, which is one of three possible final stable states (alongside polarization and fragmentation).

Herding has traditionally been studied in various markets worldwide, in institutional funds and even among financial analysts. However, interest in this behaviour has recently increased following the emergence of various cryptocurrencies, mainly focusing on price imitation between cryptocurrencies (Bouri et al. 2019; da Gamma Silva et al. 2019; Kallinterakis and Wang 2019; Stavroyiannis and Babalos 2019; Vidal-Tomás et al. 2019; Ballis and Drakos 2020; Kaiser and Stöckl 2020; Kyriazis 2020; Philippas et al. 2020; Raimundo Júnior et al. 2020; Yarovaya et al. 2021). The information sequences among different cryptocurrencies may have peculiar characteristics compared with stocks, bonds or other currencies, especially due to not only the underlying spirit and technology of this new type of asset but also other spot market indicators such as liquidity, scalability, and the lack of official recognition. All these features can modify the expected reaction of investors. Corbet et al. (2019) summarize the main characteristics of cryptocurrencies and their role as an alternative investment and as a source of diversification while recognizing some correlation between specific markets at specific times.

Although sharing a common base, current cryptocurrencies may show distinct levels of liquidity and generate different degrees of trust among investors. For this reason, for the purpose of this paper, we prefer to focus on a single cryptocurrency, bitcoin, which represented approximately 75% of the market capitalization of the 10 top cryptocurrencies in January 2021.^1^ By doing so, we intend to isolate our results from other effects that can be associated with other cryptocurrencies with their own characteristics.

Similar to the opening and closing of any trading session, the expiration date has been identified as an information-revealing time. Spot markets that have an associated derivatives market are supposed to be more complete since they enable a wider range of arbitrage and hedging strategies, even price manipulation. On the expiration date, investors must decide about their market position, either closing and settling, rebalancing or rolling over, based on their information and expectations. In fact, the so-called witching hour (the last hour of trading when options and futures contracts expire) is often characterized by heavy volumes as traders close out or roll their positions before expiry. In turn, such decisions generate new information. Furthermore, some authors, such as Kumar and Seppi (1992), point out that price manipulation is more intense around expiration dates, which leads to uncertainty and increases risk. All these extra information flows revealing sophisticated investors’ strategies, added to the habitual information sets that also affect non-expiration dates, can noticeably foster mimetic behaviour among investors. The effects of these informational changes on market variables such as returns, volume and volatility have been studied in depth (Stoll and Whaley (1987, 1991) or Alkbäkc and Hagelin (2004), among others), but it seems to be clear that this information flow could affect investors’ reactions and, in particular, their mimetic behaviour when making their decisions. To date, however, this approach has been little studied.

Cryptocurrency markets are not exempt from these effects on futures expiration dates. Since December 2017, when the Chicago Board Options Exchange (CBOE) and CME started trading regulated bitcoin futures, investors have shown active involvement in this market,^2^ and therefore, their strategies may have induced herding effects in the bitcoin spot market around expiration dates. In light of these arguments, we propose to test the following working hypotheses:

### Hypothesis 1

There is a herding effect among bitcoin exchanges before the bitcoin futures expiration date.

### Hypothesis 2

If it exists, herding disappears after the futures expiration date.

**Fig. 1:**
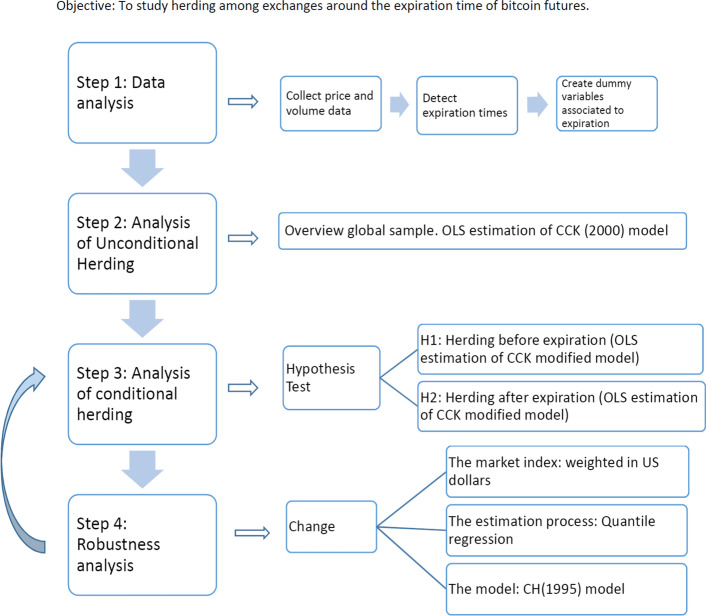
Flow chart of the analysis process

Figure 1 shows a flow chart with the main steps of the analysis and the procedures that we followed, including some robustness tests.

## Database

Bitcoin futures contracts traded in regulated markets began their journey in December 2017. Within only one week of each other, the CBOE and the CME launched their respective futures contracts (on 10 and 17 December, respectively), although the CBOE stopped trading bitcoin futures in summer 2019. Shortly thereafter, in September 2019, the Intercontinental Exchange (ICE) introduced a new bitcoin futures contract traded on the Bakkt platform.

To consider the largest possible number of observations, we focus on the futures contracts offered by the CME, which remained uniform during the period of analysis and registered the highest volume of all three regulated markets. The expiration time of this futures contract takes place on the last Friday of each month at 4:00 p.m. London time, and the settlement price is based on the Bitcoin Reference Rate (BRR) calculated by the CME. The BRR is a daily reference index that aggregates the bitcoin quotes of major spot exchanges to ensure credibility. These constituent exchanges are Bitstamp, Coinbase, itBit and Kraken (since December 2017) as well as Gemini (since 30 August 2019). The contract unit (contract multiplier) is 5 bitcoins, the price quotation is expressed in US dollars and cents per bitcoin, and the settlement method is cash settlement.

For the purpose of this paper, we take hourly bitcoin prices from seven reference exchanges. Specifically, Bitstamp, Coinbase, itBit, Kraken and Gemini, the constituent exchanges of the CME, are chosen because they provide information that can be applied to compute the price of the underlying asset in CME bitcoin futures. We also include Binance and Bitfinex since they are clear references in terms of trading volume. All of these exchanges are usually among the top 10% of bitcoin exchanges.

The data source is http://www.cryptodatadownload.com, which offers hourly closing prices and trading volumes in bitcoin and US dollars. Unix Timestamp is taken to unify the time information of all the markets under analysis, as it is based on UTC, is nearly monotonic, and is easier to parse and use across different operating systems and file formats. Daylight savings time is also considered when the Unix Timestamp is converted into human-readable local time.

Given that the aim of this paper is to analyse herding among exchanges around expiration dates and given that bitcoin futures contracts started in December 2017, our database extends from December 2017 to October 2020.^3^

Table 1 shows some descriptive statistics of the bitcoin exchanges.^4^ Binance is by far the largest exchange, considering the trading volume of the different cryptocurrencies, derivatives, stable coins and tokens globally traded, followed by Coinbase, Kraken, Bitfinex and Bitstamp. In our sample, Gemini and itBit are the smallest exchanges. However, regarding bitcoin trading exclusively, Binance and Bitfinex are the most noteworthy exchanges by volume, followed by Coinbase and Bitstamp. Furthermore, the CME-constituent exchanges hold the highest scores on transparency and security items, proving to be valuable for the purpose of CME price calculation. Finally, the geographical distribution of the headquarters and registration offices of all the exchanges analysed, as well as the geographical distribution of their electronic platforms and services, allow worldwide coverage of bitcoin trading, which is also valuable for the robustness of our results.

**Table 1:** Descriptives of exchanges

Name	Country/Region	Global trading volume	T–S score	Bitcoin trading volume	
		USD		BTC	USD
Binance	Malta (started in China)	19.96 B	70.25	46,043.67	390.84 M
Bitfinex	Hong Kong and British Virgin Islands	730.63 M	71.57	18,103.00	144.84 M
Bitstamp	Luxembourg and U.K	516.52 M	80.54	9,235.25	76.43 M
Coinbase	U.S.A	2.34 B	85.31	13,157.30	111.54 M
Gemini	U.S.A	185.47 M	82.87	3,032.04	24.51 M
itBit	U.S.A	17.24 M	75.60	2,090.58	14.67 M
Kraken	U.S.A	1.33 B	75.86	6,277.87	50.54 M

Global trading volume (USD) includes the daily trading volume of all cryptocurrencies in the exchange. Bitcoin trading volume shows mean daily bitcoin trading volume for the period under analysis in BTC and USD. T–S Score shows the score of transparency and security of the exchanges

## Methodology

### Unconditional herding behaviour

One of the approaches commonly used to detect mimetic behaviour is the CCK model. This model tests the nonlinear relationship between the cross-sectional dispersion of asset returns and market returns. Herding is detected when this relationship is significantly negative. We adopt and adapt this proposal considering the cross-sectional dispersion of the prices provided by the seven exchanges under analysis and their weighted market returns. The weighting process is initially carried out by bitcoin volume.

The initial model is as follows:

1 $$CSAD = \gamma _{0} + \gamma _{1} \left| {Rm_{t} } \right| + \gamma _{2} Rm_{t}^{2} + \varepsilon _{t}$$

where CSAD_t_ represents the cross-sectional absolute deviation of bitcoin returns among the exchanges included in our sample at hour t. It is calculated as follows:

2 $${CSAD}_{t}=\frac{\sum_{1}^{n}\left|{R}_{it}-{Rm}_{t}\right|}{n}$$

where R_it_ is the hourly return of bitcoin in exchange i and R_mt_ is the hourly weighted return index of bitcoin prices. Notably, the calculation of this market return involves a subjective component, given that there is no other hourly reference index during the time horizon of the analysis. Nevertheless, as the exchanges considered are representative of the global market, we think that the market index is appropriate for our purposes. The model also includes five lags of the cross-sectional absolute deviation (CSAD) to correct for autocorrelation.^5^ The omission of relevant variables could cause the herding coefficients to be falsely significant and therefore lead to misleading results. Correcting for autocorrelation prevents herding, if detected, from being attributed to omitted variables. We adopt the ordinary least squares (OLS) procedure of estimation using Newey–West heteroskedasticity and autocorrelation consistent (HAC) covariance estimators.

The test is based on the assumption that in the presence of herding, a large movement of market returns will induce a nonlinear reduction in the CSAD measure if the exchanges involved mimic one another. Such a reaction will be reflected in a significantly negative γ_2_ coefficient.

### Herding behaviour conditioned by the expiration time

To test herding behaviour around expiration times, we extend the model by following the proposal of Zhou and Anderson (2013). In this extension, the model is conditioned by the specific event of bitcoin futures expiration. We include in the model a dummy variable D_exp_, which identifies a particular time interval associated with the expiration time. The structure of the model is described as follows:

3 $$CSAD_{t} = \gamma _{0} + \gamma _{1} D_{{\exp }} \left| {Rm_{t} } \right| + \gamma _{2} (1 - D_{{\exp }} )\left| {Rm_{t} } \right| + \gamma _{3} D_{{\exp }} Rm_{t}^{2} + \gamma _{4} (1 - D_{{\exp }} )Rm_{t}^{2} + \varepsilon _{t}$$

where variable D_exp_ is defined differently in each estimation and varies to reveal the temporal evolution before and after the expiration time according to the suggestions in Corredor et al. (2001) for computing cumulative effects. Thus, D_exp_ is substituted by D_0_, D_1pre_, D_2pre_, …D_24pre_ and D_1post_, D_2post_…D_24post_ alternatively in each regression. D_0_ takes the value of 1 for the hour of expiration and 0 otherwise, D_1pre_ takes the value of 1 both for the hour of expiration and one hour beforehand and 0 otherwise and so on until D_24pre_, which identifies the hour of expiration and 24 h beforehand. Similarly, we create dummy variables for after futures maturity, from D_1post_, which takes the value of 1 for one hour after expiration and 0 otherwise, to D_24post_, which takes the value of 1 for up to 24 h after expiration and 0 otherwise. These variables capture the effect under analysis 24 h before and after futures maturity (which may even correspond to different dates depending on the time zones).

Furthermore, very intense informational flows around the expiration week have been detected, and these flows can cause significant changes in some trading measures in more traditional markets (see, among others, the classic works of Stoll and Whaley (1987 and 1991) and Alkbäkc and Hagelin (2004), who summarize the main studies on this subject). This evidence suggests that the expiration effect might be extended to longer-than-one-day periods. In fact, the previously mentioned concept of the “witching hour” can be extended to the so-called “quadruple witching hour” if the maturities of several derivatives coincide. The “quadruple witching hour” is often linked to abnormal volumes and returns on the days around expiration. In the bitcoin market, there is a range of different futures contracts, both regulated and unregulated, and many of them have an expiration date close to the expiration of the CME futures contract, which we identify as the most relevant. The possibility of a “multiple witching hour” could make changes in investor behaviour last more than one day.

Taking into account these circumstances, we also create additional dummy variables for up to 150 h before and after the expiration time. These additional variables are intended to detect the possible herding effect approximately 5 days before and after expiration (more or less one working week before expiration, as expiration takes place on the last Friday of the month, and until halfway through the week after expiration, which includes the following weekend and three working days).

To detect herding around futures expiration, the γ_3_ estimates should be negative and significant. γ_4_ reflects the herding effect, if any, in periods not identified as expiration times. The estimation procedure involves 300 OLS regressions using Newey–West HAC covariance estimators.

## Empirical results

Table 2 shows the results of the unconditional hourly herding estimates. The coefficient associated with herding is significant and positive; therefore, we do not find evidence of herding when the global period is analysed without considering the specific expiration event. This result is consistent with the findings of Blasco and Corredor (2021) for large exchanges using daily data. In light of these first no-herding (in fact anti-herding) results, confirmed using intraday data and evidencing that investors generally react independently without following the market consensus, we think that it is interesting to take one step forward and test whether investors change their reactions and herd around the specific event of futures expiration with intraday data, which can be more revealing.

**Table 2:** Unconditional hourly herding

	Intercept	|Rm|	Rm^2^	R-squared
Coefficient	0.000094^***^	0.022284^***^	0.119712^***^	0.51
*p-value*	*0.00*	*0.00*	*0.00*	

The table shows the estimates of the following model: $CSAD_{t} = \gamma _{0} + \gamma _{1} \left| {Rm_{t} } \right| + \gamma _{2} Rm_{t}^{2} + \varepsilon _{t}$ Estimation includes five lags of CSAD. Results using Newey–West heteroscedasticity and autocorrelation consistent estimators. ^***^, ^**^, ^*^ indicate significance at 1%, 5% and 10% respectively.

Table 3 offers a summary overview of the results of the 150 estimates performed, including in the model the dummy variables related to the range of periods before the expiration time and at the expiration time itself. Each row contains the estimated parameters of the model for one dummy variable associated with the expiration time (D_0_, D_1pre_…or D_150pre_). The table shows detailed information for the nearest hours before expiration and a summary every 12 h until the farthest moments.

**Table 3:** Conditional hourly herding around the expiration. *Effects before expiration*

	|Rm| D_exp_	***p-value***	|Rm| (1 − D_exp_)	***p-value***	*Rm*^*2*^* Dexp*	***p-value***	*Rm*^2^ (1 − D_exp_)	***p-value***
D_0pre_	0.008933	*0.38*	0.022401^***^	*0.00*	− 0.057812	*0.66*	0.120766^***^	*0.00*
D_1pre_	0.024648^**^	*0.03*	0.022420^***^	*0.00*	− 0.252231	*0.12*	0.121037^***^	*0.00*
D_2pre_	0.024000^***^	*0.00*	0.022430^***^	*0.00*	− 0.243977^**^	*0.04*	0.120966^***^	*0.00*
D_3pre_	0.018397^***^	*0.00*	0.022483^***^	*0.00*	− 0.172351^*^	*0.09*	0.120649^***^	*0.00*
D_4pre_	0.016822^***^	*0.00*	0.022500^***^	*0.00*	− 0.146958^*^	*0.10*	0.120484^***^	*0.00*
D_5pre_	0.019882^***^	*0.00*	0.022492^***^	*0.00*	− 0.191643^**^	*0.03*	0.120556^***^	*0.00*
D_6pre_	0.018545^***^	*0.00*	0.022535^***^	*0.00*	− 0.187597^**^	*0.02*	0.120374^***^	*0.00*
D_7pre_	0.018927^***^	*0.00*	0.022567^***^	*0.00*	− 0.200714^**^	*0.01*	0.120217^***^	*0.00*
D_8pre_	0.022140^***^	*0.00*	0.022542^***^	*0.00*	− 0.241857^***^	*0.00*	0.120390^***^	*0.00*
D_9pre_	0.024896^***^	*0.00*	0.022526^***^	*0.00*	− 0.282716^***^	*0.00*	0.120546^***^	*0.00*
D_10pre_	0.024738^***^	*0.00*	0.022528^***^	*0.00*	− 0.279675^***^	*0.00*	0.120526^***^	*0.00*
D_11pre_	0.023111^***^	*0.00*	0.022568^***^	*0.00*	− 0.258277^***^	*0.00*	0.120264^***^	*0.00*
D_12pre_	0.022020^***^	*0.00*	0.022582^***^	*0.00*	− 0.239787^***^	*0.00*	0.120124^***^	*0.00*
D_24pre_	0.025626^***^	*0.00*	0.022651^***^	*0.00*	− 0.218656^**^	*0.02*	0.120094^***^	*0.00*
D_36pre_	0.023554^***^	*0.00*	0.022903^***^	*0.00*	− 0.210605^**^	*0.02*	0.118640^***^	*0.00*
D_48pre_	0.022224^***^	*0.00*	0.023259^***^	*0.00*	− 0.198201^***^	*0.00*	0.119003^***^	*0.00*
D_60pre_	0.023577^***^	*0.00*	0.023463^***^	*0.00*	− 0.218285^***^	*0.00*	0.118233^***^	*0.00*
D_72pre_	0.022907^***^	*0.00*	0.023513^***^	*0.00*	− 0.165259^***^	*0.00*	0.120910^***^	*0.00*
D_84pre_	0.022228^***^	*0.00*	0.023666^***^	*0.00*	− 0.153775^***^	*0.00*	0.119602^***^	*0.00*
D_96pre_	0.021132^***^	*0.00*	0.024086^***^	*0.00*	− 0.139471^***^	*0.00*	0.116873^***^	*0.00*
D_108pre_	0.021542^***^	*0.00*	0.024208^***^	*0.00*	− 0.134961^***^	*0.00*	0.118085^***^	*0.00*
D_120pre_	0.021739^***^	*0.00*	0.024560^***^	*0.00*	− 0.134089^***^	*0.00*	0.117738^***^	*0.00*
D_132pre_	0.022085^***^	*0.00*	0.024600^***^	*0.00*	− 0.129278^***^	*0.00*	0.117071^***^	*0.00*
D_144pre_	0.020183^***^	*0.00*	0.024257^***^	*0.00*	− 0.009304	*0.94*	0.115029^***^	*0.00*
D_150pre_	0.020410^***^	*0.00*	0.024261^***^	*0.00*	− 0.013052	*0.92*	0.115047^***^	*0.00*

The table shows the estimates of Eq. (3) including five lags of CSAD $CSAD_{t} = \gamma _{0} + \gamma _{1} D_{{\exp }} \left| {Rm_{t} } \right| + \gamma _{2} (1 - D_{{\exp }} )\left| {Rm_{t} } \right| + \gamma _{3} D_{{\exp }} Rm_{t}^{2} + \gamma _{4} (1 - D_{{\exp }} )Rm_{t}^{2} + \varepsilon _{t}$

D_exp_ is the dummy variable, defined differently, that takes value 1 in specific times around expiration and 0 otherwise. Each raw contains the estimated parameters of the model for one dummy variable associated to D_exp_. For example, D_0pre_ is the dummy variable that takes value 1 at the expiration hour and 0 otherwise; the dummy variable D_1pre_ takes a value of 1 both at the hour of expiration and one hour beforehand and 0 otherwise; the dummy variable D_2pre_ takes a value of 1 at the hour of expiration and 2 h beforehand and 0 otherwise and so on, until D_150pre_ which takes a value of 1 at the hour of expiration and 150 h beforehand and 0 otherwise. Results using Newey–West heteroscedasticity and autocorrelation consistent estimators. ^***^, ^**^, ^*^ indicate significance at 1%, 5% and 10% respectively

The results indicate that at both the expiration time and one hour before, there is no significant herding effect. Nevertheless, this effect is significantly noticeable from two hours before maturity, and it extends not only up to 24 h (one day) before expiration but also up to 137 h before maturity, before which the herding parameter is no longer significant. This change in significance can be appreciated by comparing the D_132pre_ and D_144pre_ results.

According to these results, Hypothesis 1 can be confirmed since our findings reveal a significant herding effect in the working week prior to the expiration time. Informational changes around expiration induce changes in investors’ behaviour, as their reaction goes from making decisions on their own to imitating each other, probably due to the uncertainty generated around expiration by the information overload hampering investors’ decision-making. Herding may seem to be a suitable alternative for making apparently informed decisions when investors cannot manage the informational excess.

Table 4 shows the same type of information but, in this case, for dummy variables associated with a number of hours after futures expiration. The results indicate that there is no herding effect in the nearest hours after expiration. However, herding appears from 7 h up to 12 h after expiration, probably because investors actively re-open new positions and generate a new, albeit briefer, informational excess that encourages mimetic behaviour. From that time, in line with the results for non-expiration days and the unconditional results, herding disappears. The joint reading of the results indicates that the herding effect starts decreasing 13 h after expiration and subsequently turns into anti-herding behaviour.

**Table 4:** Conditional hourly herding around the expiration. *Effects after expiration*

	|Rm| D_exp_	***p-value***	|Rm| (1 − D_exp_)	***p-value***	Rm^2^ D_exp_	***p-value***	Rm^2^ (1 − D_exp_)	***p-value***
D_1post_	0.012322	*0.68*	0.022285^***^	*0.00*	0.418478	*0.84*	0.119694^***^	*0.00*
D_2post_	0.021159	*0.20*	0.022292^***^	*0.00*	− 0.734098	*0.57*	0.119624^***^	*0.00*
D_3post_	0.026065^*^	*0.06*	0.022308^***^	*0.00*	− 1.355730	*0.18*	0.119485^***^	*0.00*
D_4post_	0.028366	*0.00*	0.022313^***^	*0.00*	− 0.637224^**^	*0.01*	0.119528^***^	*0.00*
D_5post_	0.010699	*0.42*	0.022175^***^	*0.00*	1.724394	*0.25*	0.120379^***^	*0.00*
D_6post_	0.007434	*0.60*	0.022172^***^	*0.00*	1.638034	*0.25*	0.120386^***^	*0.00*
D_7post_	0.039646	*0.00*	0.022354^***^	*0.00*	− 0.588967^**^	*0.01*	0.119911^***^	*0.00*
D_8post_	0.040108^***^	*0.00*	0.022342^***^	*0.00*	− 0.591755^**^	*0.01*	0.120019^***^	*0.00*
D_9post_	0.043976^***^	*0.00*	0.022336^***^	*0.00*	− 0.564799^***^	*0.00*	0.120599^***^	*0.00*
D_10post_	0.037778^***^	*0.00*	0.022282^***^	*0.00*	− 0.285752^**^	*0.04*	0.122907^***^	*0.00*
D_11post_	0.035776^***^	*0.00*	0.022309^***^	*0.00*	− 0.265037^**^	*0.05*	0.122797^***^	*0.00*
D_12post_	0.033322^***^	*0.00*	0.022336^***^	*0.00*	− 0.231733^*^	*0.06*	0.122568^***^	*0.00*
D_24post_	0.044162^***^	*0.00*	0.021857^***^	*0.00*	− 0.201611	*0.34*	0.123800^***^	*0.00*
D_36post_	0.030587^***^	*0.00*	0.022035^***^	*0.00*	− 0.005767	*0.97*	0.122074^***^	*0.00*
D_48post_	0.030284^***^	*0.00*	0.022165^***^	*0.00*	− 0.073014	*0.54*	0.123255^***^	*0.00*
D_60post_	0.028028^***^	*0.00*	0.022224^***^	*0.00*	− 0.039580	*0.74*	0.122706^***^	*0.00*
D_72post_	0.026360^***^	*0.00*	0.022313^***^	*0.00*	− 0.023231	*0.85*	0.122173^***^	*0.00*
D_84post_	0.015260^***^	*0.03*	0.022182^***^	*0.00*	0.381875	*0.16*	0.110753^***^	*0.00*
D_96post_	0.015689^***^	*0.03*	0.022224^***^	*0.00*	0.346543	*0.20*	0.111515^***^	*0.00*
D_108post_	0.015253^***^	*0.03*	0.022297^***^	*0.00*	0.349972	*0.19*	0.110961^***^	*0.00*
D_120post_	0.014833^***^	*0.02*	0.022386^***^	*0.00*	0.353606	*0.18*	0.110248^***^	*0.00*
D_132post_	0.014554^***^	*0.02*	0.022519^***^	*0.00*	0.346307	*0.18*	0.109699^***^	*0.00*
D_144post_	0.013743^***^	*0.03*	0.022783^***^	*0.00*	0.344795	*0.18*	0.108177^***^	*0.00*
D_150post_	0.013665^***^	*0.03*	0.022873^***^	*0.00*	0.340398	*0.18*	0.107759^***^	*0.00*

The table shows the estimates of Eq. (3) including five lags of CSAD$CSAD_{t} = \gamma _{0} + \gamma _{1} D_{{\exp }} \left| {Rm_{t} } \right| + \gamma _{2} (1 - D_{{\exp }} )\left| {Rm_{t} } \right| + \gamma _{3} D_{{\exp }} Rm_{t}^{2} + \gamma _{4} (1 - D_{{\exp }} )Rm_{t}^{2} + \varepsilon _{t}$

D_exp_ is the dummy variable, defined differently, that takes value 1 in specific times around expiration and 0 otherwise. Each raw contains the estimated parameters of the model for one dummy variable associated to D_exp_. For example, the dummy variable D_1post_ takes a value of 1 one hour after expiration and 0 otherwise; the dummy variable D_2post_ takes a value of 1 two hours after expiration and 0 otherwise and so on, until D_150post_ which takes a value of 1 150 h after expiration and 0 otherwise. Results using Newey–West heteroscedasticity and autocorrelation consistent estimators. ^***^, ^**^, ^*^ indicate significance at 1%, 5% and 10% respectively

These results confirm Hypothesis 2. Although some herding appears after expiration, it does not last for long and supposedly gives rise to the anti-herding reaction that basically holds until the next expiration week.

Taken together, the results lead us to conclude that there exists a differential reaction of investors around bitcoin futures expiration as opposed to non-expiration times.

**Fig. 2:**
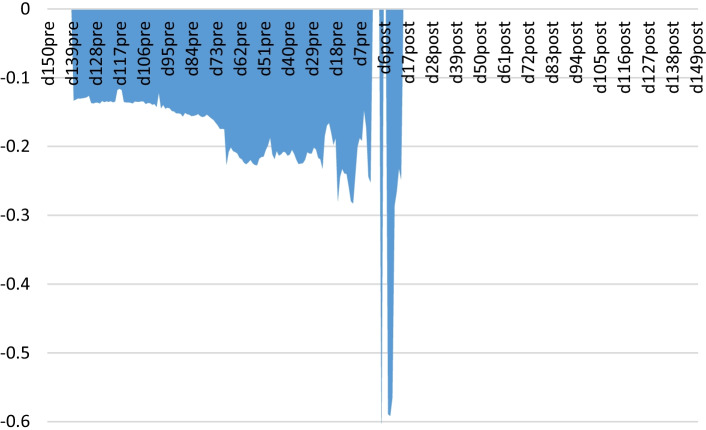
Estimates of significant herding coefficients around futures expiration

Figure 2 shows how the significant herding coefficients obtained with the regression procedure evolve over time. When bitcoin futures contracts are coming to an end, investors who trade in different exchanges mimic each other. This herding behaviour also occurs a few hours after expiration, probably due to the readjustment of strategies that takes place after expiration and the re-opening of contracts that will expire at a later date. Outside of the hours close to expiration, investors do not significantly imitate each other. In fact, we generally observe a clear anti-herding behaviour.

Regarding the concept of the witching hour, the trading volume of the exchanges that belong to our sample grows by approximately 2% at the beginning of the expiration week, reaching an increase of approximately 5.5% in the 24 h prior to expiration. These increases are corrected, at a similar pace, after expiration. The volume increases prior to expiration are consistent with the excess of information at those times (and, therefore, with the difficulty of processing it) and with the incentive of herding practices that disappear after maturity.

## Robustness analyses

The results reported above come from model estimations using a market index return weighted by bitcoin volume. To ensure the robustness of the results, the models are re-estimated using a market return calculated as an index weighted by the US volume of traded bitcoins. Table 5 shows a summary of the coefficients strictly associated with herding behaviour conditioned by the expiration time. These results are similar to those previously described, which allows us to confirm that the results do not depend on the index considered as the market reference.

**Table 5:** Robustness tests OLS regressions using the US volume to compute the return of the market index

	Before expiration				After expiration			
	Rm^2^ D_exp_	***p-value***	Rm^2^ (1-D_exp_)	***p-value***	Rm^2^ D_exp_	***p-value***	Rm^2^ (1-D_exp_)	***p-value***
D_0_	− 0.053979	*0.67*	0.130189^***^	*0.00*				
D_1_	− 0.250259	*0.11*	0.130464^***^	*0.00*	0.388974	*0.85*	0.129033^***^	*0.00*
D_2_	− 0.250472^**^	*0.03*	0.130403^***^	*0.00*	− 0.769407	*0.57*	0.128967^***^	*0.00*
D_3_	− 0.176641^*^	*0.08*	0.130074^***^	*0.00*	− 1.432647	*0.17*	0.128811^***^	*0.00*
D_4_	− 0.148310^*^	*0.10*	0.129895^***^	*0.00*	− 0.643818^**^	*0.01*	0.128847^***^	*0.00*
D_5_	− 0.194445^**^	*0.03*	0.129967^***^	*0.00*	1.680901	*0.26*	0.129735^***^	*0.00*
D_6_	− 0.191027^**^	*0.02*	0.129779^***^	*0.00*	1.579844	*0.27*	0.129743^***^	*0.00*
D_7_	− 0.209440^**^	*0.01*	0.129641^***^	*0.00*	− 0.616743^**^	*0.01*	0.12927^***^	*0.00*
D_8_	− 0.251632^***^	*0.00*	0.129823^***^	*0.00*	− 0.613975^**^	*0.01*	0.129371^***^	*0.00*
D_9_	− 0.293610^***^	*0.00*	0.129984^***^	*0.00*	− 0.580343^***^	*0.00*	0.129977^***^	*0.00*
D_10_	− 0.289650^***^	*0.00*	0.129958^***^	*0.00*	− 0.287979^**^	*0.05*	0.132318^***^	*0.00*
D_11_	− 0.269501^***^	*0.00*	0.129698^***^	*0.00*	− 0.266867^*^	*0.06*	0.132208^***^	*0.00*
D_12_	− 0.250075^***^	*0.00*	0.12955^***^	*0.00*	− 0.23315^*^	*0.07*	0.131977^***^	*0.00*
D_24_	− 0.217145^**^	*0.02*	0.129434^***^	*0.00*	− 0.223279	*0.34*	0.133618^***^	*0.00*
D_36_	− 0.210673^**^	*0.02*	0.127982^***^	*0.00*	− 0.003387	*0.98*	0.131698^***^	*0.00*
D_48_	− 0.200823^***^	*0.00*	0.128431^***^	*0.00*	− 0.07922	*0.52*	0.133018^***^	*0.00*
D_60_	− 0.222531^***^	*0.00*	0.127654^***^	*0.00*	− 0.041301	*0.74*	0.132417^***^	*0.00*
D_72_	− 0.168169^***^	*0.00*	0.130481^***^	*0.00*	− 0.024072	*0.85*	0.13192^***^	*0.00*
D_84_	− 0.156841^***^	*0.00*	0.129151^***^	*0.00*	0.395257	*0.16*	0.120234^***^	*0.00*
D_96_	− 0.142887^***^	*0.00*	0.126423^***^	*0.00*	0.370497	*0.19*	0.120421^***^	*0.00*
D_108_	− 0.139415^***^	*0.00*	0.127735^***^	*0.00*	0.373491	*0.18*	0.119851^***^	*0.00*
D_120_	− 0.141292^***^	*0.00*	0.127462^***^	*0.00*	0.376754	*0.17*	0.119123^***^	*0.00*
D_132_	− 0.139337^***^	*0.00*	0.126884^***^	*0.00*	0.36893	*0.17*	0.118529^***^	*0.00*
D_144_	− 0.015067	*0.90*	0.124704^***^	*0.00*	0.366243	*0.17*	0.116988^***^	*0.00*
D_150_	− 0.018918	*0.88*	0.124733^***^	*0.00*	0.361358	*0.17*	0.116555^***^	*0.00*

The table shows the estimates of Eq. (3) including five lags of CSAD$CSAD_{t} = \gamma _{0} + \gamma _{1} D_{{\exp }} \left| {Rm_{t} } \right| + \gamma _{2} (1 - D_{{\exp }} )\left| {Rm_{t} } \right| + \gamma _{3} D_{{\exp }} Rm_{t}^{2} + \gamma _{4} (1 - D_{{\exp }} )Rm_{t}^{2} + \varepsilon _{t}$

D_exp_ is the dummy variable, defined differently, that takes value 1 in specific times around expiration and 0 otherwise. Each raw contains the estimated parameters of the model for one dummy variable associated to D_exp_. For example, the dummy variable D_1_ takes a value of 1 one hour before (after) expiration and 0 otherwise; the dummy variable D_2_ takes a value of 1 2 h before (after) expiration and 0 otherwise and so on, until D_150_ that takes a value of 1 150 h before (after) expiration and 0 otherwise. Results using Newey–West heteroscedasticity and autocorrelation consistent estimators. ^***^, ^**^, ^*^ indicate significance at 1%, 5% and 10% respectively

To provide additional alternative estimates not based on the mean, we use the quantile regression procedure. This methodology allows the model to be estimated in different quantiles of a distribution. We use the 50th quantile of the conditional distribution of the CSAD, which is representative of the median of the distribution. Table 6 lists the main coefficients for the bitcoin volume weighted index.^6^ The results obtained allow us to confirm the previous findings since the herding effect is observed in the hours before expiration, although it starts slightly later (approximately 112 h before expiration) than when using other regression methods. The herding effect after expiration occurs during a shorter period and then, consistent with our previous findings, gives rise to significant anti-herding behaviour.

**Table 6:** Robustness tests using quantile regressions

	Before expiration				After expiration			
	Rm^2^ D_exp_	***p-value***	Rm^2^ (1 − D_exp_)	***p-value***	Rm^2^ D_exp_	***p-value***	Rm^2^ (1 − D_exp_)	***p-value***
D_0_	− 0.013186	*0.91*	0.106122^***^	*0.00*				
D_1_	− 0.142918	*0.29*	0.113435^***^	*0.00*	0.981784	*0.23*	0.107023^***^	*0.00*
D_2_	− 0.170789^***^	*0.00*	0.113514^***^	*0.00*	0.782161	*0.34*	0.107138^***^	*0.00*
D_3_	− 0.085632	*0.25*	0.112832^***^	*0.00*	− 0.043864	*0.97*	0.107066^***^	*0.00*
D_4_	− 0.089831	*0.11*	0.112729^***^	*0.00*	− 0.099513	*0.79*	0.106936^***^	*0.00*
D_5_	− 0.085813	*0.15*	0.111250^***^	*0.00*	0.367007	*0.78*	0.107200^***^	*0.00*
D_6_	− 0.055984	*0.29*	0.112948^***^	*0.00*	0.661392	*0.59*	0.107234^***^	*0.00*
D_7_	− 0.055442	*0.33*	0.111882^***^	*0.00*	− 0.425801^***^	*0.00*	0.106638^***^	*0.00*
D_8_	− 0.090121^**^	*0.02*	0.112287^***^	*0.00*	− 0.410641^***^	*0.00*	0.106639^***^	*0.00*
D_9_	− 0.128446^*^	*0.05*	0.113445^***^	*0.00*	− 0.337711	*0.39*	0.106667^***^	*0.00*
D_10_	− 0.128106^**^	*0.02*	0.113516^***^	*0.00*	0.034026	*0.53*	0.114780^***^	*0.00*
D_11_	− 0.097982^**^	*0.01*	0.112689^***^	*0.00*	0.044743	*0.18*	0.113423^***^	*0.00*
D_12_	− 0.089636^**^	*0.02*	0.112217^***^	*0.00*	0.080440	*0.27*	0.113600^***^	*0.00*
D_24_	− 0.128187^***^	*0.00*	0.113447^***^	*0.00*	0.035516	*0.23*	0.107183^***^	*0.00*
D_36_	− 0.098464^***^	*0.00*	0.112197^***^	*0.00*	0.059866^***^	*0.00*	0.105874^***^	*0.00*
D_48_	− 0.087003^***^	*0.00*	0.110951^***^	*0.00*	0.060119^***^	*0.00*	0.113675^***^	*0.00*
D_60_	− 0.083854^***^	*0.00*	0.110769^***^	*0.00*	0.058194^***^	*0.00*	0.113647^***^	*0.00*
D_72_	− 0.054904	*0.84*	0.111672^***^	*0.00*	0.056161^***^	*0.00*	0.114722^***^	*0.00*
D_84_	− 0.045361	*0.85*	0.110657^***^	*0.00*	0.059461^***^	*0.00*	0.113765^***^	*0.00*
D_96_	− 0.078991^***^	*0.00*	0.109752^***^	*0.00*	0.060059^***^	*0.00*	0.113441^***^	*0.00*
D_108_	− 0.081603^***^	*0.00*	0.109956^***^	*0.00*	0.059868^***^	*0.00*	0.113845^***^	*0.00*
D_120_	− 0.035914	*0.88*	0.110315^***^	*0.00*	0.059316^***^	*0.00*	0.113899^***^	*0.00*
D_132_	− 0.040240	*0.86*	0.110850^***^	*0.00*	0.061740^***^	*0.00*	0.113383^***^	*0.00*
D_144_	0.005853	*0.61*	0.111669^***^	*0.00*	0.063808^***^	*0.00*	0.111452^***^	*0.00*
D_150_	0.004261	*0.71*	0.112111^***^	*0.00*	0.064497^***^	*0.00*	0.110977^***^	*0.00*

The table shows the estimates of Eq. (3) including five lags of CSAD $CSAD_{t} = \gamma _{0} + \gamma _{1} D_{{\exp }} \left| {Rm_{t} } \right| + \gamma _{2} (1 - D_{{\exp }} )\left| {Rm_{t} } \right| + \gamma _{3} D_{{\exp }} Rm_{t}^{2} + \gamma _{4} (1 - D_{{\exp }} )Rm_{t}^{2} + \varepsilon _{t}$

D_exp_ is the dummy variable, defined differently, that takes value 1 in specific times around expiration and 0 otherwise. Each raw contains the estimated parameters of the model for one dummy variable associated to D_exp_. For example, the dummy variable D_1_ takes a value of 1 one hour before (after) expiration and 0 otherwise; the dummy variable D_2_ takes a value of 1 2 h before (after) expiration and 0 otherwise and so on, until D_150_ that takes a value of 1 150 h before (after) expiration and 0 otherwise. Results using Newey–West heteroscedasticity and autocorrelation consistent estimators. ^***^, ^**^, ^*^ indicate significance at 1%, 5% and 10% respectively

Finally, we also conduct a specific robustness analysis using one of the frequently referenced proposals in the financial literature: the seminal model by Christie and Huang (1995) (CH). The CH model tests the linear relationship between the dispersion of returns and extreme market returns and detects herding under extreme market movements. An initial analysis of our sample indicates that approximately 98.7% of the 1% extreme positive returns and the 1% extreme negative returns of our period of analysis fall within non-expiration periods. This result means that the CH model is not the best method for detecting herding around the expiration time. Nevertheless, the CH model may be useful for testing what happens outside expiration times and, in particular, for checking the anti-herding behaviour detected on non-expiration days as well as the significantly positive linear relationships shown in the first two columns of Tables 3 and 4 using the CCK model.

Table 7 summarizes the main results. The first two rows present the result of the average estimates and the significance of the variables included in the model around expiration times and at non-expiration times. As expected, the anti-herding behaviour on non-expiration days is clearly detected, as is a positive linear relationship between dispersion measures and market returns, although this relationship is not strictly significant at the usual 10% confidence level. Compared with the first two columns of Tables 3 and 4, which are based on the CCK model, we think that the results differ for two reasons: first, because the linear relationship in the CCK model considers both extreme and non-extreme market returns and, second, because of the possibility of herding at very specific periods with extreme returns. For this reason, we also include in the table (third and fourth rows) the average estimates (and average significance levels) corresponding to the expiration variables D_4pre_ to D_8pre_ and D_8post_ to D_11post_. On the one hand, consistent with our initial findings, with the CH model, we detect herding starting 8 h before expiration until 4 h before expiration in the case of the lowest market returns. On the other hand, we find negative coefficients, although not significant, during the same post-expiration hours as in the CCK model. Once again, it is important to remember that the CH model detects herding only at times of extreme returns. These returns seldom occur around futures maturity times.

**Table 7:** Robustness tests using the CH(1995) model

	Extreme 1% lower market returns				Extreme 1% larger market returns			
	D^L^D_exp_	***p-value***	D^L^(1 − D_exp_)	***p-value***	D^U^D_exp_	***p-value***	D^L^(1 − D_exp_)	***p-value***
Aver. D_0pre_ − D_150pre_	0.0004	*0.10*	0.0017	*0.00*	0.0004	*0.11*	0.0011	*0.00*
Aver. D_1post_ − D_150post_	0.0007	*0.12*	0.0017	*0.00*	0.0005	*0.18*	0.0010	*0.00*
Aver. D_4pre_ − D_8pre_	− 0.0003	*0.00*	0.0016	*0.00*	0.0003	*0.21*	0.0010	*0.00*
Aver. D_8post_ − D_11post_	0.0001	*0.59*	0.0016	*0.00*	− 2.9E−05	*0.63*	0.0010	*0.00*

The table shows the estimates of the CH(1995) equations including five lags for the dispersion of returns $CSSD_{t} = \alpha _{0} + \beta _{{11}} D_{{\exp }} D_{t}^{L} + \beta _{{12}} (1 - D_{{\exp }} )D_{t}^{L} + \beta _{{21}} D_{{\exp }} D_{t}^{U} + \beta _{{22}} (1 - D_{{\exp }} )D_{t}^{U} + \varepsilon _{t}$

D_exp_ is the dummy variable, defined differently, that takes value 1 in specific times around expiration and 0 otherwise. D^L^ = 1 if the market return at time t lies in the 1% extreme lower tail of the return distribution and 0 otherwise. D^U^ = 1 if the market return at time t lies in the 1% extreme upper tail of the return distribution and 0 otherwise. In the table: Aver. D_4pre_ − D_8pre_ is the variable representing the average of D_exp_ estimates (from D_4pre_ to D_8pre_) and their average significance in parentheses (from D_4pre_ to D_8pre_); Aver. D_0pre_ − D_150pre_ is the variable representing the average of D_exp_ estimates (from D_0pre_ to D_150pre_) and their average significance in parentheses (from D_0pre_ to D_150pre_); Aver. D_8post_ − D_11post_ is the variable representing the average of D_exp_ estimates (from D_8post_ to D_11post_) and their average significance in parentheses (from D_8post_ to D_11post_) and Aver. D_1post_ − D_150post_ is the variable representing the average of D_exp_ estimates (from D_1post_ to D_150post_) and their average significance in parentheses (from D_1post_ to D_150post_)

In summary, the CH model supports our previous findings confirming the anti-herding behaviour on non-expiration days and the noticeable linear relationship between dispersion measures and extreme market returns. It even suggests the relevance of the herding effect some hours prior to the expiration time when extreme negative returns appear. Hence, bearing in mind the differences between the CH and CCK models, we conclude that the findings of the CH test are consistent with our initial results.

In this robustness analysis, we used some of the common approaches to detect herding. However, some authors have recently proposed analytical tools using models coming from physics and mathematics to address financial issues. Kou et al. (2014) and Li et al. (2021) show the usefulness of some multi-criteria decision-making (MCDM) methods and adaptive algorithms for evaluating clustering algorithms and detecting clusters in financial data. In the same vein, Kou et al. (2021a) use fuzzy methodology, and Kou et al. (2021b) propose multi-objective optimization. Zha et al. (2020) indicate that some binary opinion dynamics models could help in understanding the decision-making process. As a social phenomenon, herding behaviour can be affected by multiple latent factors. In the future, research on this behaviour could also be framed as a multi-criteria and/or clustering problem around an event.

## Conclusions

The purpose of this paper is to test the herding effect among bitcoin exchanges around the expiration date of bitcoin futures. The emergence of bitcoin futures contracts in regulated markets offers a unique opportunity to analyse the mimetic behaviour of investors more closely. Bitcoin futures have attracted interest from institutional investors who consider this new asset an additional investment opportunity. This interest helps to increase the liquidity of the spot market and leads to the appearance of sophisticated investors in Bitcoin markets. If these investors seek to take advantage of the expiration time in their speculative or arbitrage strategies, less informed investors will probably monitor and imitate their movements.

Using intraday data and an unconditional model, we confirm, on average, anti-herding behaviour for the period. However, our results go one step further by showing that at certain moments, in which the discovery of strategies and information overload are key features, herding may appear if investors find difficulties in processing information to generate their expectations. Consistent with our hypotheses, we find a strong herding effect before expiration and a few hours after expiration. Specifically, these effects extend throughout the week prior to expiration and disappear quickly the day after expiration.

The results suggest that bitcoin prices generally reflect investors’ own information, but particularly in the week of bitcoin futures expiration, this point is questionable since investors seem to watch each other closely. If the herding effect is evident during the expiration week, the information flow may not be as informative and could be contaminated.

The herding effect should be studied in detail since, as our findings indicate, it is not homogeneous at all times. The influence of important factors such as fear of missing out (FOMO), confirmation bias and overconfidence (see, among others, Merkle and Weber (2011) or Baur and Dimpfl (2018)) on the psychology of investors that causes the decision to herd means that herding is important enough to be analysed conditioned by the occurrence of different events and in various markets. In addition, the strong growth in bitcoin trading is itself the result of psychological factors that make this analysis even more interesting. Furthermore, as Corbet et al. (2019) point out, there must be ongoing research on cryptocurrencies since their behaviour is continually changing.

In general, the direct consequences of herding for financial investors occur in two ways. On the one hand, herding makes it more difficult to diversify investment portfolios, and on the other hand, financial assets may be mispriced due to price pressures that increase volatility and market instability and that can therefore drive prices away from their expected values. In our paper, the first consequence does not have great implications for relevant investors since we analyse the herding that occurs between exchanges that trade the same asset, bitcoin, and, presumably, their diversification strategies mainly focus on different assets instead of the same asset in different markets or on different platforms. However, mispricing can affect all investors in all exchanges at maturity times, with the exception of bitcoin holders (hodlers), whose objective is long-term gains.

Around maturity time, investors are aware of the number of informational elements that may influence decision-making. For this reason, many uninformed investors may find it useful to imitate the decisions of others, transferring that imitation to the different exchanges where bitcoin is traded. Herding between exchanges can amplify the mispricing of bitcoin since imitation spreads throughout different markets and platforms. Consequently, market players will not be able to adequately predict prices and may find volatility levels that intensify the risk assumed. In volatile markets such as crypto markets and at times of volatility, price slippages tend to occur. Therefore, at the time of executing a transaction during herding periods, asset prices can shift noticeably before the transaction is completed. Hence, exchanges should ensure that liquidity providers such as market makers (makers) and liquidity pools create multiple bid-ask orders to match the orders (especially large orders) of other traders to execute transactions instantaneously and to reduce price slippages.

More specifically, our results also have implications for investors who design their strategies encompassing several exchanges since they must take into account that price differentials narrow in the hours close to expiration. Consequently, market participants who act as arbitrageurs or hedgers betting on different exchanges in bitcoin will have more limited possibilities of obtaining profits. Arbitrage traders who participate in triangular arbitrage trading (which involves spotting the price differences between three different cryptocurrencies, even on the same exchange) should review their strategies involving bitcoin, while hedging and arbitrage investors operating in both the spot and futures markets should review their hedge ratios.

Policy makers should also realize that the expiration week is an atypical week in which exchanges tend to follow the market consensus. This behaviour could be worrying in the case of large market fluctuations when the risk associated with feelings of pessimism or euphoria could spread to all exchanges.

In financial markets, decision-making is a significant issue. Herding is a consequence of decision-making, which is why it is interesting to understand it from different perspectives. In the future, to further investigate investors’ behaviour in financial markets, as Zha et al. (2020) suggest, it will be necessary to conduct integrated in-depth interdisciplinary research.

## Data Availability

The datasets analysed during the current study are clearly detailed in the text of the paper. Basically: http://www.cryptodatadownload.com, https://www.cryptocompare.com/exchanges/#/overview, https://coinmarketcap.com/es/rankings/exchanges/

## Footnotes

1 The data are provided by the CME Group and coinmarketcap.com.

2 As of 20 May 2021, Arcane Research reports a new trading record ($200B) considering regulated and unregulated bitcoin futures contracts.

3 The end of the period is driven by the data availability in http://www.cryptodatadownload.com.

4 The global trading volume is the average of the information provided by https://www.cryptocompare.com/exchanges/#/overview and https://coinmarketcap.com/es/rankings/exchanges/ on 14 June 2021 and includes all assets traded. The T-S Score represents the sum (over 100) of some category items such as regulation, data provision, security, trade monitoring and the negative reports provided by CryptoCompare (https://www.cryptocompare.com/exchanges/#/overview, June 14^th^ 2021).

5 To avoid the serial autocorrelation detected, the number of lags is jointly determined by their significance (lags 6 and 7 were not significant), the closest-to-2 values of the Durbin–Watson (DW) statistic and the minimum values of the Akaike information criterion (AIC).

6 The results using the US volume weighted index are similar and are available upon request from the authors.
